# Multidrug-resistant *Staphylococcus pettenkoferi* isolated from cat in India

**DOI:** 10.14202/vetworld.2018.1380-1384

**Published:** 2018-10-05

**Authors:** Tapan Kumar Dutta, Satyaki Chakraborty, Malay Das, Rajkumari Mandakini, Vanrahmlimphuii, Parimal Roychoudhury, Santanu Ghorai, Suvendu Kumar Behera

**Affiliations:** 1 Department of Veterinary Microbiology, Central Agricultural University, Selesih, Aizawl - 796 014, Mizoram, India; 2 Department of Veterinary Public Health and Epidemiology, Central Agricultural University-Imphal, Selesih, Aizawl - 796 014, Mizoram, India; 3 Department of Veterinary Microbiology, Central Agricultural University-Imphal, Jalukie, Nagaland-India; 4 Department of Veterinary Medicine, Central Agricultural University-Imphal, Selesih, Aizawl - 796 014, Mizoram, India

**Keywords:** cat, India, multidrug resistance, *Staphylococcus pettenkoferi*

## Abstract

**Background and Aim:**

Coagulase-negative staphylococci (CoNS) are considered to be one of the emerging pathogens in human and animals in recent times. *Staphylococcus pettenkoferi*, a novel pathogen under CoNS, is discovered in 2002 in humans with multiple clinical manifestations in various patients. To date, the pathogens have not yet been reported from any animals. The present study reported the first ever isolation, identification, and characterization of multidrug-resistant *S. pettenkoferi* from a cat with peritonitis in India.

**Materials and Methods:**

Peritoneal fluid was collected aseptically from 3 years old cat processed for bacteriological culture by standard techniques. Isolates were confirmed by BD Phoenix™ automated bacterial identification system and were subjected to plate and tube coagulase tests. All the isolates were tested for antimicrobial sensitivity profile by disc diffusion assay, extended-spectrum β-lactamase production by double disc diffusion assay, *in vitro* biofilm production ability by microtiter plate assay, and detection of virulence genes and *mecA* gene by polymerase chain reaction assay.

**Results:**

A total of five clonally expanded isolates of *S. pettenkoferi* were isolated from peritoneal fluid of the affected cat. All the isolates were resistant against 36 antimicrobial agents and were also methicillin-resistant staphylococci. Phenotypically, all the isolates were negative for biofilm production but were carrying multiple biofilm-producing genes (*icaA*, *IS257*, *nuc*, and *mecA)*.

**Conclusion:**

Although *S. pettenkoferi* was previously reported once from animal (cat) environment, this is probably the first ever report of isolation of the organism directly from any animals. This is also probably the first report from any species in India.

## Introduction

The genus *Staphylococcus*, one of the most common and ubiquitous Gram-positive bacteria worldwide, comprises more than 40 species and subspecies. The coagulase-negative staphylococci (CoNS) are considered one of the most common pathogenic agents of nosocomial bacteremia throughout the world [1]. Although, the CoNS are low-virulence pathogens but are frequently contaminate specimens obtained from non-sterile and sterile sites of patients [1]. *Staphylococcus pettenkoferi* is one of the new members of the CoNS family first reported by Hashi *et al*. [2] from blood culture of an extrapulmonary tuberculosis patient and also from wound of patient with leukemia, cancer, and insulin-dependent diabetes mellitus. The same group again could isolate *S*. *pettenkoferi* from different patients also [2]. Since then, there are few sporadic reports on the association of *S*. *pettenkoferi* as a causative agent of osteomyelitis with diabetes, blood infection of hospitalized patients, and other immunocompromised patients [1-7]. In the recent past, Weiss *et al*. [8] reported two isolates of *S. pettenkoferi* from a feeding dish and blanket in a pet cage of a small animal clinic. However, to date, there is no published report available on isolation of *S*. *pettenkoferi* from animals in any country. In addition, it has also not been reported from any human patients in India.

The present study reported the first ever isolation, identification, and characterization of multidrug-resistant *S. pettenkoferi* from a cat with peritonitis in India.

## Materials and Methods

### Ethical approval

The research work was conducted with the permission of Institutional Animal Ethics Committee, College of Veterinary Sciences & Animal Husbandry, CAU(I), Selesih, Aizawl, Mizoram.

### Isolation and identification

Peritoneal fluid collected aseptically with a sterile syringe from a 3-year-old cat with symptoms of ascites received from Teaching Veterinary Clinical Complex, College of Veterinary Sciences and Animal Husbandry, Central Agricultural University, Aizawl, Mizoram, India, was inoculated on 5% sheep blood agar plate and incubated at 37°C for 24 h. The pure bacterial colonies (n=5) were stained by Gram’s method, and each colony was subjected to confirmation of bacterial species by BD Phoenix^™^ system as per the manufacturer’s instruction. All the five bacterial colonies were recovered as *S. pettenkoferi*. All the isolates were subjected to plate and tube coagulase tests and recorded as negative.

### Antimicrobial sensitivity tests

Antimicrobial susceptibility test was done on Mueller–Hinton agar (MHA) plate as per the recommendation of the Clinical Laboratory Standard Institute [9] using 43 commercially available antibiotic discs (HiMedia, India) (Table-1). *Escherichia coli* ATCC 25922 and *Salmonella* Enteritidis ATCC 13076 were used as control organisms.

**Table-1:** Details of antimicrobial sensitivity pattern of five isolates of *S. pettenkoferi* recovered from peritoneal fluid of cat in India.

Antimicrobials	Family	Sensitivity status
Penicillin-G (10)	Narrow spectrum beta-lactamase	Resistant
Methicillin (30)	Narrow spectrum beta-lactamase	Resistant
Cloxacillin (30)	Narrow spectrum beta-lactamase	Resistant
Ampicillin (30)	Broad spectrum beta-lactamase	Resistant
Ampicillin (10)	Broad spectrum beta-lactamase	Resistant
Amoxicillin (30)	Broad spectrum beta-lactamase	Resistant
Cephalothin (30)	First-generation cephalosporin	Resistant
Cefuroxime (30)	Second-generation cephalosporin	Resistant
CAZ (30)	Third-generation cephalosporin	Resistant
CTX (30)	Third-generation cephalosporin	Intermediate
CPD (10)	Third-generation cephalosporin	Resistant
Ceftriaxone (30)	Third-generation cephalosporin	Resistant
CTX/clavulanate (30/10)	Third-generation cephalosporin	Resistant
Neomycin (30)	Aminoglycoside	Resistant
Streptomycin (25)	Aminoglycoside	Resistant
Amikacin (30)	Broad spectrum aminoglycoside	Intermediate
Gentamycin (10)	Extended-spectrum aminoglycoside	Sensitive
Furazolidone (50)	Nitrofuran	Resistant
Nitrofurantoin (300)	Nitrofuran	Resistant
Enrofloxacin (10)	Fluoroquinolone	Sensitive
Ciprofloxacin (5)	Fluoroquinolone	Resistant
Norfloxacin (10)	Fluoroquinolone	Sensitive
Levofloxacin (5)	Fluoroquinolone	Sensitive
Lomefloxacin (10)	Fourth-generation fluoroquinolone	Intermediate
Erythromycin (15)	Macrolide	Resistant
Oleandomycin (15)	Macrolide	Resistant
Clarithromycin (15)	Macrolide	Intermediate
Azithromycin (15)	Semisynthetic macrolide	Resistant
Doripenem (10)	Carbapenem	Resistant
Meropenem (10)	Carbapenem	Sensitive
Imipenem (10)	Carbapenem	Sensitive
Lincomycin (10)	Lincosamide	Resistant
Clindamycin (10)	Lincosamide	Resistant
Sulfafurazole (300)	Short-acting sulfa drug	Resistant
Sulfadiazine (300)	Short-acting sulfonamides	Resistant
Trimethoprim (5)	Sulfa drug	Intermediate
Co-trimoxazole (25)	Broad spectrum sulfa drug	Intermediate
Tetracycline (30)	Broad spectrum tetracycline	Sensitive
Chloramphenicol (10)	Chloramphenicol	Resistant
ATZ (30)	Monobactam	Resistant
Novobiocin (30)	Aminocoumarin	Resistant
Vancomycin (30)	Glycopeptide	Resistant
Bacitracin (10)	Polypeptide	Intermediate

Figures in parenthesis are the mg of antimicrobials in each disc. *S. pettenkoferi*=*Staphylococcus* *pettenkoferi*, CTX=Cefotaxime, CAZ=Ceftazidime, ATZ=Aztreonam, CPD=Cefpodoxime

### Phenotypic screening for evaluation of extended-spectrum β-lactamase (ESBL) production

All the confirmed *S. pettenkoferi* isolates were screened for their minimum inhibitory concentration (MIC) against cefotaxime (CTX), ceftazidime (CAZ), and aztreonam (ATZ). The isolates with MIC of ≥2 µg/ml were further screened for ESBL production by double disk synergy test (DDST), combination disk method (CDM), and broth microdilution method (BMM). In DDST, the test inoculum with 0.5 McFarland turbidity was streaked into MHA; HiMedia, India and disks of CTX, CAZ, ATZ, cefepime, and cefpodoxime (30 µg each) were manually placed at 20 mm distance center to center surrounding a disk of amoxicillin-clavulanate (AMC; 20/10 µg). Following overnight incubation at 37°C, the test isolate was considered positive, if its zone of inhibition against any of the peripheral disk was enhanced toward AMC [10]. In case of CDM, an increase of 5 mm or more in zone diameter for CAZ/CTX in combination with clavulanic acid (CA) than that of the corresponding cephalosporin disk was interpreted as ESBL positive. In BMM, Mueller-Hinton broth containing serial two-fold dilution of CTX and CAZ was prepared at different concentrations ranging from 0.25 to 512 µg/ml, with or without fixed concentration of CA (4 µg/ml), and inoculated with the confirmed isolates in a 96-well microplate. Following overnight incubation, at 37°C, a minimum of three-fold decrease in the MICs of CTX and or CAZ in combination with CA versus CTX and/or CAZ alone was considered as positive for ESBL production.

### Detection of in vitro biofilm production ability of the bacterial isolates

Biofilm assay was performed by microtiter plate biofilm assay as described by Månsson *et al*, [11]. In brief, both the isolates were grown in lysogeny broth (LB) supplemented with 0.9% NaCl and 1% glucose and incubated at 37°C for 24 h under constant shaking at 130 rpm. The cultures were diluted at 1:50 in fresh LB broth supplemented with 0.9% NaCl and 1% glucose to make a final concentration of 1×10^7^ colony-forming unit/200 µl and inoculated in three consecutive wells of 96-well tissue culture plates including negative control. The plate was incubated at 37°C for 24 h without shaking. After washing by phosphate-buffered saline (pH-7.4) and fixing with 99% methanol, each well was stained with 0.1% aqueous crystal violet. Excess stain was gently rinsed off with tap water, and the plate was air dried. Finally, the stain was solubilized in 200 µL of 95% ethanol with shaking in an orbital shaker for 30 min and the OD_595_ values were determined in a microplate reader.

### Detection of virulence genes and mecA genes by polymerase chain reaction (PCR)

Both the isolates were subjected to PCR for detection of *icaA*, *icaD, IS257, nuc, TSST1, coa*, and *mecA* genes as described earlier [12]. The amplified products were visualized by gel documentation system (UVP, UK) after electrophoresis in 1.5% (W/V) agarose gel containing ethidium bromide (0.5 µg/mL) (Merck, Germany).

## Results

As mentioned earlier, a total of five identical colonies were recovered from the blood agar plate. All the colonies were 1-2 mm in diameter, circular, smooth, slightly convex, glistening, and opaque with entire margins. There was no pigmentation after 2 days of incubation. All the colonies were Gram-positive cocci with the typical staphylococcal arrangement. All the isolates were catalase positive and coagulase negative. The isolates were identified and confirmed as *S. pettenkoferi* by BD Phoenix^™^. All the five isolates were also recorded as negative for both free coagulase and bound coagulase.

All the five isolates were recorded as negative for biofilm production by *in vitro* microtiter plate biofilm assay. By PCR, all the isolates were found to be positive for *icaA*, *IS257*, *nuc*, and *mecA* genes but negative for *icaD*, *coa*, and *TSST 1* genes.

Among the 43 antimicrobial agents used for *in vitro* antimicrobial sensitivity assay, all the five isolates exhibited resistance against 36 agents and were sensitive only against gentamycin, fluoroquinolones (enrofloxacin, norfloxacin, and levofloxacin), carbapenems (imipenem and meropenem), and tetracycline (Table-1). The isolates were resistance against all narrow spectrum and broad-spectrum β-lactam antimicrobial agents including methicillin. The isolates were also found to be resistant against all the cephalosporin group of antimicrobial agents including third-generation cephalosporins. The isolates were also resistance against glycopeptides (vancomycin), aminocoumarin (novobiocin), as well as polypeptides (bacitracin). None of the isolates were found to be positive for ESBL production by DDST or CDM or BMM.

## Discussion

In the present study, the *S. pettenkoferi* isolates were confirmed by BD Phoenix™ system (Becton, Dickinson and Company, USA) based on the inbuilt 46 biochemical and sugar fermentation test profile. Previously, all the workers reported that the existence of *S. pettenkoferi* from various sources was confirmed by basic biochemical tests, sugar fermentation tests, as well as 16S rRNA sequencing followed by BLAST analysis [1-8]. So far, no worker reported on the identification of *S. pettenkoferi* using this bacterial identification system. For further confirmation, all the isolates were screened for 16S rRNA based PCR and confirmed as *S. pettenkoferi*. Our finding could be a positive validation for the use of Phoenix bacterial identification system for confirmation of bacterial species. All the five isolates under this study were CoNS, which is in corroboration with previous workers [1-8].

The *S. pettenkoferi* isolates were found to be positive for *icaA*, *IS257*, and *nuc* and negative for *icaD*, *coa*, and *TSST1* genes. *icaA* and *icaD* are two important biofilm-associated signature genes which encode the proteins mediating the synthesis of polysaccharide intercellular adhesin PIA and polysaccharide-adhesin PS/A in staphylococcal species and helps in the colonization of bacteria [12]. However, the study isolates were recorded as negative for biofilm formation tested by *in vitro* microtiter plate assay. Earlier, Vasudevan *et al*. [13] also reported the high prevalence of *ica* genes among *Staphylococcus aureus* mastitis isolates, which were not always been associated with *in vitro* formation of slime or biofilm. The *IS257* genes have also been implicated in biofilm formation. Moreover, *IS257* is a mobile genetic element, which confers resistance to aminoglycoside and beta-lactam antibiotics [14]. To survive in the host, staphylococci secrete the array of tissue degrading enzymes, toxins, and superantigens. The secreted nuclease (Nuc) is an enzyme, which has both endonuclease and exonuclease properties and can degrade both RNA and DNA [15]. Other signature toxins of *S. aureus* such as *TSST-1* and *coa* help to increase pathogenicity during infection. Toxic shock syndrome is a rare condition associated with menstruating women using tampons and is characterized by rapid onset of fever and multi-organ failure [15]. Phenotypically, all the isolates under the present study were resistant to methicillin. The methicillin-resistant staphylococci (MRS), particularly the MR *S. aureus* (MRSA), are considered to be a major public health concern. Detection of *mecA* gene in the isolates further confirmed its resistance against methicillin. The first report of *S. pettenkoferi* by Loïez *et al*. [6] did not highlight the resistance against methicillin. Song *et al*. [7] reported resistance against cloxacillin and confirmed by detection of *mecA* gene but not tested for methicillin. *mecA* gene is considered as a marker for MRSA, and hence, detection of *mecA* gene confirmed the isolates as resistant against methicillin.

The most striking observation under the present study is the multidrug resistance of the *S. pettenkoferi* isolates. As depicted in Table-1, all the isolates were resistant against 36 of 43 antimicrobial agents. Teeraputon *et al*. [10] reported that the MSSA isolates are sensitive to most of the antibiotics tested, whereas the MRSA is multiple drug resistance and is sensitive against amikacin, erythromycin, and norfloxacin. In the present study, *S. pettenkoferi* isolates showed resistant to methicillin and another β-lactamase group of antimicrobials but sensitive to norfloxacin. Detection of methicillin resistance generally helps to predict resistance to other classes of antimicrobials besides beta-lactams. In all the previous reports [1-8], *S. pettenkoferi* isolates were described as sensitive to novobiocin and vancomycin. However, our isolates were resistant to novobiocin, vancomycin, as well as bacitracin. In addition to that, the isolates were also resistant to doripenem, a carbapenem group of new generation of antibiotic. It is reported that *S. pettenkoferi* used to present in the indoor environment and confirmed by direct analysis of the 16S rRNA gene from settled dust samples [16]. The organisms might have acquired the multiple drug resistance through environmental sources in this region, but at the same time, so far no incidence of human infection due to such MDR organisms was reported. Therefore, the possibility of contraction of such organisms by the infected animal through environment may be considered. In addition, as all the five colonies recovered from the peritoneal fluid showed identical characters, they might have clonally expanded and were the same strain.

Due to the lack of complete study on other probable causative agents, it could not be concluded that the *S. pettenkoferi* isolated from the peritoneal fluid of the cat is the causative agent of peritonitis. Although Weiss *et al*. [8] reported two isolates of *S. pettenkoferi* from the cat environment (one from the blanket and another from feeding dish) those were not directly from cats of the same clinic. So far, many researchers reported *S. pettenkoferi* from various clinical conditions of human patients, which established the role of the organism as a human pathogen. Both the isolates of *S. pettenkoferi* reported by Weiss *et al*. [8] might be from human or cats of the same clinic but not confirmed further. In this regard, our present findings may be considered as the first-ever report on isolation of *S. pettenkoferi* directly from any animals. To the best of our knowledge, it is also the first ever report of *S. pettenkoferi* from any host species in India. In addition, it can also be stated that detection of such novel organism with a high level of resistant against major antimicrobial agents is probably a serious public health concern. A comprehensive detail epidemiological study on detection, characterization, and antibiotic resistance pattern of *S. pettenkoferi* in man and animals in various geographical regions of India requires to be undertaken to understand the possible threat to public health.

## Conclusion

*S. pettenkoferi* is not a known animal pathogen and not being isolated directly from any animals so far. Although, it was previously reported once from animal (cat) environment, this is probably the first ever attempt for isolation of the organism directly from any animals. This is also probably the first report from any species in India.

## Authors’ Contributions

TKD: Planning of the work, preparation of the manuscript. SC: Isolation and identification of organisms. MD: Antimicrobial resistance study. RM: Detection of virulence genes and antibiofilm genes. V: Phenotypic detection of biofilm production ability of the isolates. PR: Analysis of data and PCR. SG: Collection of samples and clinical diagnosis of the affected animal. SKB: Collection of literature and preparation of the manuscript. All authors read and approved the final manuscript.
